# Synaptic biomarkers in the cerebrospinal fluid associate differentially with classical neuronal biomarkers in patients with Alzheimer’s disease and frontotemporal dementia

**DOI:** 10.1186/s13195-023-01212-x

**Published:** 2023-03-24

**Authors:** Shreyasee Das, Julie Goossens, Dirk Jacobs, Nele Dewit, Yolande A. L. Pijnenburg, Sjors G. J. G. In ‘t Veld, Charlotte E. Teunissen, Eugeen Vanmechelen

**Affiliations:** 1 ADxNeuroSciences NV, Zwijnaarde 94, 9052 Ghent, Belgium; 2 Neurochemistry Laboratory, Department of Clinical Chemistry, Amsterdam Neuroscience, Program Neurodegeneration, Amsterdam UMC, Vrije Universiteit Amsterdam, De Boelelaan 1117, 1081 HV Amsterdam, The Netherlands; 3 Medpace, Technologielaan 11, 3001 Leuven, Belgium

**Keywords:** Biomarkers, Immunoassay, Synapse, Dementia, Alzheimer’s disease, Frontotemporal dementia

## Abstract

**Background:**

Loss of synaptic functionality has been recently identified as an early-stage indicator of neurological diseases. Consequently, monitoring changes in synaptic protein levels may be relevant for observing disease evolution or treatment responses in patients. Here, we have studied the relationship between fluid biomarkers of neurodegeneration and synaptic dysfunction in patients with Alzheimer’s disease (AD), frontotemporal dementia (FTD), and subjective cognitive decline (SCD).

**Methods:**

The exploratory cohort consisted of cerebrospinal fluid (CSF) samples (*n* = 60) from patients diagnosed with AD (*n* = 20), FTD (*n* = 20), and SCD (*n* = 20) from the Amsterdam Dementia Cohort. We developed two novel immunoassays for the synaptic proteins synaptosomal-associated protein-25 (SNAP25) and vesicle-associated membrane protein-2 (VAMP2). We measured the levels of these biomarkers in CSF, in addition to neuronal pentraxin-2 (NPTX2), glutamate ionotropic receptor-4 (GluR4), and neurogranin (Ng) for this cohort. All in-house immunoassays were validated and analytically qualified prior to clinical application. CSF neurogranin (Ng) was measured using a commercially available ELISA.

**Results:**

This pilot study indicated that SNAP25, VAMP2, and Ng may not be specific biomarkers for AD as their levels were significantly elevated in patients with both AD and FTD compared to SCD. Moreover, the strength of the correlations between synaptic proteins was lower in the AD and FTD clinical groups compared to SCD. SNAP25, VAMP2, and Ng correlated strongly with each other as well as with total Tau (Tau) and phosphorylated Tau (PTau) in all three clinical groups. However, this correlation was weakened or absent with NPTX2 and GluR4. None of the synaptic proteins correlated to neurofilament light (NfL) in any clinical group.

**Conclusion:**

The correlation of the synaptic biomarkers with CSF Tau and PTau but the lack thereof with NfL implies that distinct pathological pathways may be involved in synaptic versus axonal degeneration. Our results reflect the diversity of synaptic pathology in neurodegenerative dementias.

**Supplementary Information:**

The online version contains supplementary material available at 10.1186/s13195-023-01212-x.

## Background

Alzheimer’s disease (AD) and frontotemporal dementia (FTD) are two of the most prevalent causes of dementia among the elderly. AD is a slow-progressing neurodegenerative disorder that is primarily characterized by memory and cognitive impairments while FTD is a largely heterogeneous disorder that manifests as behavioral changes, language impairment, motor symptoms, or even psychiatric disorders [1–3]. The pathological signature of AD is amyloid beta (Aβ) plaque accumulation in the neocortex of the brain, while in FTD, there is degeneration of the frontal and temporal lobes, including in para-limbic areas [4, 5].

Synaptic dysfunction and loss of synaptic plasticity are fundamental processes that underlie several neurodegenerative disorders from early stages. In fact, synaptic pathology has been identified in pre-clinical stages of AD, FTD, dementia with Lewy bodies, and Parkinson’s disease, as well as certain psychiatric disorders [5–7]. Synaptic proteins are involved in differential pathological processes in patients with AD and several of these proteins are also associated with their corresponding total Tau (Tau) and phosphorylated tau (PTau) levels in CSF [7, 8]. The diversity of synaptic pathology across several neurodegenerative dementia is further reflected by the fact that strong associations can be found between some synaptic proteins but not all, as well as the reported lack of correlation of synaptic biomarkers with CSF neurofilament light (NfL) levels [1, 9–11].

The current advancements in cerebrospinal fluid (CSF) biomarkers for neurodegenerative dementias have revolutionized the clinical approach towards these disorders, not only for their diagnostic value but also for disease prognosis and critical therapeutic interventions [12, 13]. However, the pathological interplay between loss of synaptic functionality and neurodegeneration remains poorly understood [14]. In addition to the above concern, even though classical biomarkers such as Aβ42, Tau, and PTau are accepted clinical diagnostic hallmarks of AD, they are not predictive of cognitive decline [9].

**Fig. 1:**
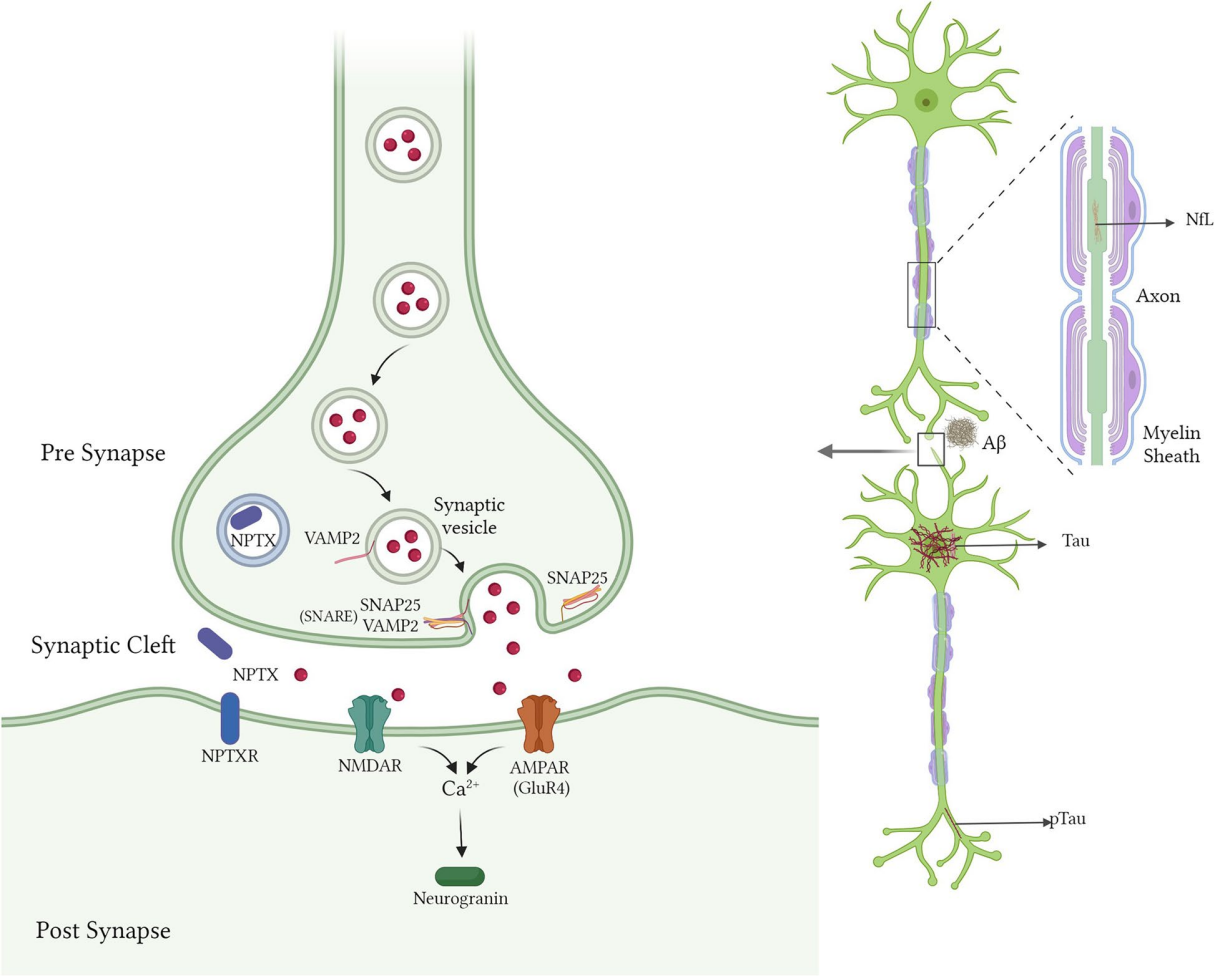
Schematic representation of the pre- and post-synaptic terminals of neurons. Aβ, amyloid beta; PTau, phosphorylated Tau (at threonine 181); NfL, neurofilament light chain; VAMP2, vesicle-associated membrane protein-2; SNAP25, synaptosomal associated protein-25; SNARE, SNAP Receptor Complex; NPTX2, neuronal pentraxin-2; GluR4, glutamate receptor-4; AMPAR, α-amino-3-hydroxy-5-methyl-4-isoxazole propionic acid receptor; NMDAR, N-methyl-D-aspartate receptor

A recent proteomic analysis of CSF revealed a panel of synaptic biomarkers that were expressed differentially through the progression of AD disease stages. This panel included glutamate ionotropic receptor-4 (GluR4) and vesicle-associated membrane protein-2 (VAMP2) [5]. VAMP2 and synaptosomal associated protein-25 (SNAP25) are presynaptic proteins, part of the soluble N-ethylmaleimide-sensitive-factor Attachment Receptor (SNARE) complex, that play a crucial role in the fusion of synaptic vesicles with the presynaptic plasma membrane to facilitate the release of neurotransmitters [15]. GluR4 is a subunit of the α-amino-3-hydroxy-5-methyl-4-isoxazole propionic acid receptor (AMPAR) complex that is present in the postsynaptic domain and is responsible for excitatory signal transmission [16]. Another biomarker, neurogranin (Ng), is a protein present in the dendritic spine that regulates calcium influxes and is found to be increased in AD [10]. A synaptic protein progressively decreasing with the disease state in genetic FTD is neuronal pentraxin-2 (NPTX2), mainly present as an extracellular protein in the synaptic cleft [7] Neuronal pentraxins, including NPTX2, are involved in synaptic plasticity, and their lower levels have been reported in patients with DLB, FTD, and PD as well [16]. The schematic representation in Fig. 1 depicts the localization of pre- and post-synaptic proteins, their receptors, and other fluid biomarkers.

We have attempted to address some of the knowledge gaps regarding synaptic pathology in AD and FTD compared to normally aged individuals with subjective cognitive decline (SCD). In this explorative cohort, we measured the synaptic proteins SNAP25, VAMP2, Ng, NPTX2, and GluR4 in clinical CSF samples of patients with AD, FTD, and SCD and studied their correlation with classical fluid biomarkers Aβ42, Tau, PTau, and NfL. Patients with both AD and FTD were included to assess the implied AD specificity of certain synaptic proteins such as SNAP25, VAMP2, and Ng [5, 9, 17]. Our primary hypotheses were as follows: (a) CSF synaptic protein levels reflect pathologies that are distinct from broad-range neuro-axonal degeneration and (b) the associations between synaptic proteins differ in the clinical disease states of AD and FTD compared to cognitively normal individuals.

Along with the evaluation of our clinical hypotheses, we also developed two novel in-house immunoassays: an ELISA for VAMP2 and a Simoa-based assay for SNAP25. Before use, these immunoassays were validated for their analytical performance following established guidelines [18].

## Materials and methods

### CSF samples and clinical cohort design

Remnant CSF samples, purchased from Biomnis, were used to perform the validations of the VAMP2 and SNAP25 in-house immunoassays. The age and gender data were available for all these CSF samples. The clinical cohort was composed of patients (*n* = 60) who were diagnosed as a part of the Amsterdam Dementia Cohort [19]. The diagnostic groups within this cohort were as follows: AD (*n* = 20), FTD (*n* = 20), and SCD (*n* = 20). For each of these patients, levels of CSF biomarkers Aβ42, Tau, and PTau their age, gender, ApoE status, and their cognitive score (mini-mental state examination, MMSE) were available via the Amsterdam Dementia Cohort Biobank. Aβ42, Tau, and PTau were measured using commercially available kits from Elecsys® (Roche Diagnostics GmbH, Penzberg, Germany) and enzyme-linked immunosorbent assay (ELISA) INNOTEST (Fujirebio, Gent, Belgium). For analytical convenience, these biomarker values were converted to INNOTEST values using established guidelines [20]. The samples were screened for their levels of CSF Aβ42, Tau, and PTau to support their AD diagnosis (Tau/Aβ42 > 0.46) [21]. The FTD diagnoses were based on consensus guidelines for FTD criteria along with psychiatric evaluations as a part of the Amsterdam Dementia Cohort [22, 23]. All FTD patients in this cohort were classified as “probable behavioral” variant. Individuals with SCD were patients who had subjective memory complaints but did not meet the clinical diagnostic criteria for AD or MCI. They were also negative for AD biomarkers. Demographic characteristics of the clinical samples used are tabulated in Table 1. All immunoassays were performed blind and the samples were randomized.

**Table 1:** Demographic characteristics of the clinical cohort

		**Diagnostic groups**					
Demographic characteristics	**AD**	**FTD**	**SCD**	**ANOVA** ***P*-value**	**Adjusted *P*-value** **SCD vs FTD**	**Adjusted *P*-value** **SCD vs AD**	**Adjusted *P*-value** **AD vs FTD**
*n*	20	20	20	-	-	-	-
Age	66 (58, 75)	64 (61, 68)	55 (51, 66)	0.02	ns	0.030	ns
Sex, female	10 (50%)	10 (50%)	5 (25%)	ns	ns	ns	ns
MMSE	21 (11, 25)	21 (17, 26)	29 (27, 30)	< 0.001	< 0.001	< 0.001	ns
Aβ42	494 (443, 604)	846 (723, 1087)	1036 (870, 1101)	< 0.001	ns	< 0.001	< 0.001
Tau	613 (516, 980)	385 (265, 482)	190 (159, 234)	< 0.001	0.003	< 0.001	0.015
PTau	83 (61, 111)	45 (38, 66)	35 (26, 42)	< 0.001	0.014	< 0.001	0.006
NfL	899 (702, 1160)	1880 (1495, 3346)	409 (292, 578)	< 0.001	< 0.001	0.006	0.006

Demographic characteristics of patients. The data for sample size is presented as *n* (%) or as median (interquartile range). Age is shown in years and the fluid biomarker values are as pg/mL. *P*-values were calculated using Kruskal–Wallis’ one-way ANOVA and post hoc Dunn’s multiple comparisons. *ns*, not significant. This table has been previously published as part of a different project [25]

### Development and validation of immunoassays for SNARE complex proteins VAMP2 and SNAP25 in CSF

Antibodies against VAMP2 were produced using hybridoma technology and phage display technology by performing peptide immunizations in OF1 mice and rabbits, respectively. The peptide immunizations and antibody reformatting were carried out at Biotem (Apprieu, France) in compliance with the EU animal welfare legislation. All mice bleeding and serum analysis experiments were performed in accordance with the ARRIVE guidelines, following good laboratory practices and ethical regulations at Biotem [24]. Five mice were immunized with four low doses (10 μg) of a short keyhole limpet hemocyanin (KLH)-coupled peptide, S27-R40, of human VAMP2 (Uniprot ID P63027). The spleen was harvested from one selected mouse and its lymphocytes were fused with Sp2/0-Ag14 myeloma cells. Fused cells were plated out in 96-well plates and culture supernatants were screened on biotinylated peptide via ELISA, and via Western Blot analysis on HEK293T cell lysate with overexpressed human VAMP2 (LY415423, Origene Technologies, Maryland, USA), and human temporal cortex extracts (Tissue Solutions, Glasgow, UK). One monoclonal antibody (mAb), 15E4, was selected and used as a detector antibody in the final ELISA format. Two rabbits were immunized with a short KLH-coupled peptide G15-L32 of human VAMP2. One rabbit was euthanized, the spleen was harvested, B cells were isolated, total RNA was extracted, and cDNA was amplified to construct a single-chain fragment variable (ScFv) library in a custom phagemid. Three rounds of phage display were performed on a long peptide (S2-Q34) and enrichment was seen in round three. Ninety-six individual clones were hand-picked and screened on the BSA-coupled long peptide and recombinant human VAMP2 (Novus Biologicals, NBC1-18335). Non-redundant clones were selected after sequencing, and two ScFvs (G11 and H12) were reformatted into full rabbit IgG antibodies and produced via a recombinant CHO DG44 expression system. The antibodies raised against VAMP2 were first checked for their specificity using the Western Blot technique against human whole-brain extracts (h181 and h1389), and a human brain synaptosome extract (h181syn) obtained from Sant Pau Memory Unit, Barcelona, Spain. We then mapped the epitopes of the antibodies using an indirect ELISA technique as described previously [25]. A sandwich ELISA was developed for detecting VAMP2 in CSF using G11 mAb as the capture antibody and 15E4 mAb as the biotinylated detector antibody.

For SNAP25, we developed a Simoa-based assay where the ADx404 antibody (Biolegend, Clone SMI.81) was used as the capture antibody coupled to paramagnetic beads and a biotinylated antibody RD042 (Clone 71.1, Synaptic Systems, Göttingen, Germany) was used as the detector. The in-house immunoassays were validated following the methods described previously [18]. The immunoassays were validated for their precision, lower limit of quantification (LLOQ), dilutional linearity, and parallelism. We set the acceptable range of deviation for dilutional linearity and parallelism at 80 to 120% and the acceptable range of variation in precision, %CV, was set at ≤ 15%. OD values that were below the LLOQ of the ELISA were not taken into consideration while analyzing the validation criteria. The precision of the immunoassays was checked by determining the intra-assay and inter-assay variability using three remnant CSF samples covering the calibrator range.

### Other immunoassays

The 60 clinical CSF samples were also measured for their concentrations of NfL, NPTX2, and GluR4 using ELISAs developed and validated in-house [25, 26]. For GluR4 measurements, a clinically validated in-house ELISA immunoassay was used [27]. Ng levels of these samples were measured with the commercially available Neurogranin Trunc p75 kit from Euroimmun (EQ6551-9601-L).

### Data analysis and statistics

All statistical analyses were performed using GraphPad Prism software (v.6.02) or IBM SPSS Statistics (v.28.0.1.1). Power calculation for the sample set was performed on G*Power (v.3.1.9.7) [28]. We performed a “compromise power analysis” for one-way ANOVA (*F*-tests) for a total sample size of 60 spread equally across 3 groups. The calculations were performed assuming a large effect size (*f* = 0.4) and the ratio of type II to type I error (β/α) equals 1. The power of the statistical test (1-β) was thus determined to be 88.1%. For the immunoassays, the calibrator points were log-transformed on both X and Y coordinates and were fitted with an unweighted sigmoidal 4-PL curve fit. Baseline demographics and clinical characteristics were compared using Kruskal–Wallis’ one-way ANOVA and post hoc Dunn’s multiple comparisons. The distribution of the biomarker concentrations was checked using Shapiro–Wilk’s normality test. The biomarker values were transformed to logarithmic scale to fit a normal distribution. A general linear model was used to correct for age and sex. Post hoc pairwise comparisons were used to determine biomarker differences between the clinical groups. For normally distributed biomarkers, Pearson’s correlation coefficient was used to study the associations between CSF biomarkers, while for non-parametric datasets, Spearman’s rank correlation coefficient was used. Receiver operating characteristics (ROC) analysis was performed on the biomarkers and the area under the curves (AUC) determined, along with confidence intervals using De Long’s test. To select a biomarker panel for each diagnostic group comparison, we used Wald’s backward logistic regression on the synaptic biomarkers. The significance for all the statistical tests was set at *P* < 0.05.

## Results

### Analytical validation of novel immunoassays

The analytical validation results of the VAMP2 ELISA and SNAP25 Simoa are summarized in Table 2. The epitope mapping and specificity testing of the antibodies used to develop these novel immunoassays are indicated in Supplementary Tables 1–2 and Supplementary Figs. 1–4.

**Table 2:** Validation results of immunoassays for VAMP2 and SNAP25

**Parameter**	**Acceptance criteria**	**VAMP2 ELISA**				**SNAP25 Simoa**			
**Sensitivity – LLOQ Conc**	Mean_blank_ + 10*SD_blank_	127 pg/ml				2.91 pg/ml			
**Precision**	CV < 15%	**Conc**	**Intra-assay**		**Inter-assay**	**Conc**	**Intra-assay**		**Inter-assay**
Low sample		235	6.3%		6.3%	4	8.3%		10.6%
Medium sample		299	2.6%		4.5%	6	14.3%		14.3%
High sample		748	3.5%		3.5%	10	7.9%		7.9%
		**Mean**	4.1%		4.8%	**Mean**	10.2%		10.9%
**Dilutional linearity**	80–120% linearity	**Mean 3 samples**				**Mean 3 samples**			
		**DF (x)**		**%L**		**DF (x)**		**%L**	
		1		-		1		-	
		2		155		3		108	
		4		107		6		112	
		8		106		16		115	
		16		107		39		99	
		32		97		98		97	
		64		143					
		**Mean**		110%		**Mean**		106%	
**Parallelism**	80–120% in range with the slope of the calibrator	**Mean 3 samples**				**Mean 5 samples**			
		**Mean**		**SD**		**Mean**		**SD**	
*R*^2^		0.94		0.0263		0.98		0.005	
Slope		1.54		0.109		1.41		0.071	
Range		103%		7%		89%		4%	

Partial validation of the immunoassays: novel VAMP2 ELISA and SNAP25 Simoa. All concentration values are in pg/mL. The table shows the LLOQ of the immunoassays; precision for 3 different CSF samples with low, medium, and high analyte concentrations as the mean %CV of samples intra-assay and inter-assay; the mean dilutional linearity for 3 CSF samples against their dilution factors; and the mean %parallelism with the calibrator for 5 CSF samples (*SD*, standard deviation; *R*^2^, linear regression coefficient). VAMP2 ELISA: The mean intra- and inter-assay precision was 4.1% and 4.8% respectively, while the mean dilutional linearity was 110%. The mean parallelism on CSF samples was 103%. SNAP25 Simoa: The mean intra- and inter-assay precision was 10.2% and 10.9% respectively, while the mean dilutional linearity was 106%. The mean parallelism on CSF samples was 89%

#### VAMP2 ELISA

The VAMP2 CSF sandwich ELISA had an LLOQ of 127 pg/mL. The mean intra-assay and inter-assay %CV were 4.1% and 4.8% respectively; thus, the assay is highly precise. The mean dilutional linearity of the three CSF samples was 110%, which was well within the acceptability criteria. Furthermore, mean parallelism of 103% with a 7% standard deviation (SD) was observed, thereby indicating minimal CSF matrix effects in the assay. Lastly, the CSF samples had a percentage recovery within 80 to 120% up to four freeze–thaw cycles, implying that the analyte remains stable with multiple freeze-thaws (Supplementary Fig. 5A).

#### SNAP25 Simoa

The SNAP25 CSF Simoa assay had an LLOQ of 2.91 pg/mL. The mean intra-assay and inter-assay %CV were 10.2% and 10.9% respectively, both being well within the accepted limit. Three CSF samples were spiked with 200 pg of calibrator protein and serially diluted up to a dilution factor of 98. The mean dilutional linearity of the samples was found to be 106%, which is within the acceptability criteria. The mean parallelism for five CSF samples was 89% which represents minimal matrix effects in the assay. A minor 0.6% negative drift effect was observed for the CSF signals after 3 h 45 min of sample incubation, indicating potential instability of the analyte upon long incubation periods (Supplementary Fig. 5B). This was mitigated by limiting the sample incubation time to 20 min and placing calibrators and run-validation samples at the beginning and end of the immunoassay well plates.

### Levels of synaptic proteins SNAP25, VAMP2, and Ng are elevated in CSF of patients with AD and FTD

As expected, the core neuronal biomarkers in CSF: Tau, PTau, and NfL, were able to differentiate significantly between the clinical groups of AD, FTD, and SCD (Supplementary Fig. 6B–D). CSF Aβ42 was significantly decreased in patients with AD compared to FTD and SCD, but could not differentiate between the clinical groups FTD and SCD (Supplementary Fig. 6A).

**Fig. 2:**
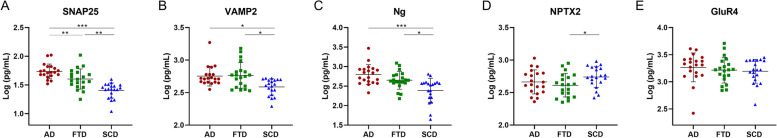
Scatter plot of the synaptic CSF fluid biomarkers in the three diagnostic groups AD (*n* = 20), FTD (*n* = 20), and SCD (*n* = 20). The biomarker values were log-transformed to fit a normal distribution and the model corrected for age and sex prior to conducting pairwise multiple comparisons. **A** SNAP25, **B** VAMP2, **C** Ng, **D** NPTX2, and **E** GluR4. *P < 0.05, **P < 0.01, ***P < 0.001

Patients in both the AD and FTD groups had significantly higher levels of CSF SNAP25, VAMP2, and Ng compared to the SCD group (Fig. 2A–C). Among these, SNAP25 levels alone were significantly elevated in AD compared to FTD. Furthermore, FTD patients had significantly lower NPTX2 levels than the SCD group (Fig. 2D). However, we did not find a differential expression of the synaptic biomarker GluR4 in any clinical group within this cohort (Fig. 2E). All patients in this cohort had CSF SNAP25, VAMP2, Ng, NPTX2, and GluR4 levels above the detection limit of the immunoassays used.

### Diagnostic potential of the synaptic biomarkers and selection of panels for differential diagnosis

**Fig. 3:**
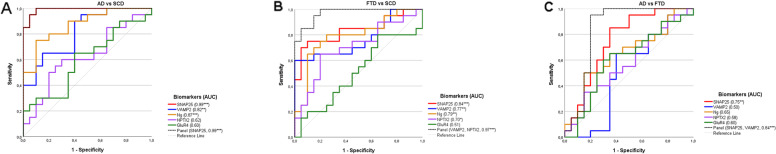
ROC curves and AUC values of the synaptic CSF biomarkers among the clinical groups. **A** AD versus SCD. **B** FTD versus SCD. **C** AD versus FTD. ROC, receiver operating characteristics, AUC, area under the curve. *P < 0.05, **P < 0.01, ***P < 0.001

We have visualized the diagnostic value of each synaptic biomarker for differentiating between the clinical group AD vs SCD, FTD vs SCD, and AD vs FTD (Fig. 3A–C; Table 3). SNAP25 (AUC = 0.99) had the greatest diagnostic value for differentiating AD vs SCD patients, followed by Ng (AUC = 0.87) and VAMP2 (AUC = 0.82). No significant AUC was observed for NPTX2 (AUC = 0.62) or GluR4 (AUC = 0.60). The biomarker panel selection consisted of SNAP25 alone (AUC = 0.99). A similar trend was observed for the biomarkers while comparing the clinical groups FTD vs SCD where the synaptic proteins had significant diagnostic values as follows: SNAP25 (AUC = 0.83), Ng (AUC = 0.79), VAMP2 (AUC = 0.77), NPTX2 (AUC = 0.70). The selected synaptic biomarker panel with the highest AUC consisted of VAMP2 and NPTX2 (AUC = 0.97). Interestingly, between the clinical group AD vs FTD, none of the synaptic biomarkers except SNAP25 (AUC = 0.75) showed significant diagnostic value. The identified biomarker panel consisted of SNAP25 and VAMP2 and had a higher AUC than the individual biomarkers (AUC = 0.84).

**Table 3:** Diagnostic value of the CSF biomarkers

	**Clinical groups**					
	**AD vs SCD**		**FTD vs SCD**		**AD vs FTD**	
**Core biomarker**	**AUC**	***P*-value**	**AUC**	***P*-value**	**AUC**	***P*-value**
Aβ42	1.00 (1.00, 1.00)	< 0.001	0.66 (0.48, 0.84)	0.079	0.92 (0.83, 1.00)	< 0.001
Tau	0.98 (0.99, 1.00)	< 0.001	0.87 (0.74, 0.99)	< 0.001	0.82 (0.69, 0.96)	< 0.001
PTau	1.00 (1.00, 1.00)	< 0.001	0.81 (0.67, 0.94)	0.001	0.83 (0.69, 0.96)	< 0.001
NfL	0.88 (0.76, 0.99)	< 0.001	0.98 (0.95, 1.00)	< 0.001	0.88 (0.75, 1.00)	< 0.001
**Synaptic biomarker**	**AUC**	***P*-value**	**AUC**	***P*-value**	**AUC**	***P*-value**
SNAP25	0.99 (0.96, 1.00)	< 0.001	0.83 (0.70, 0.96)	< 0.001	0.75 (0.58, 0.89)	0.007
VAMP2	0.82 (0.69, 0.95)	< 0.001	0.77 (0.62, 0.92)	0.003	0.50 (0.31, 0.69)	0.978
Ng	0.87 (0.77, 0.98)	< 0.001	0.79 (0.64, 0.93)	0.002	0.65 (0.48, 0.82)	0.110
NPTX2	0.62 (0.45, 0.79)	0.185	0.70 (0.54, 0.87)	0.028	0.57 (0.39, 0.76)	0.402
GluR4	0.60 (0.42, 0.78)	0.291	0.51 (0.32, 0.69)	0.935	0.60 (0.42, 0.78)	0.267
Selected panel	0.99 (0.96, 1.00)	< 0.001	0.97 (0.93, 1.00)	< 0.001	0.84 (0.70, 0.98)	< 0.001

The data is represented as AUC (95% confidence interval). *AUC* area under the curve

### Synaptic biomarkers revealed a differential association with classical fluid biomarkers

**Fig. 4:**
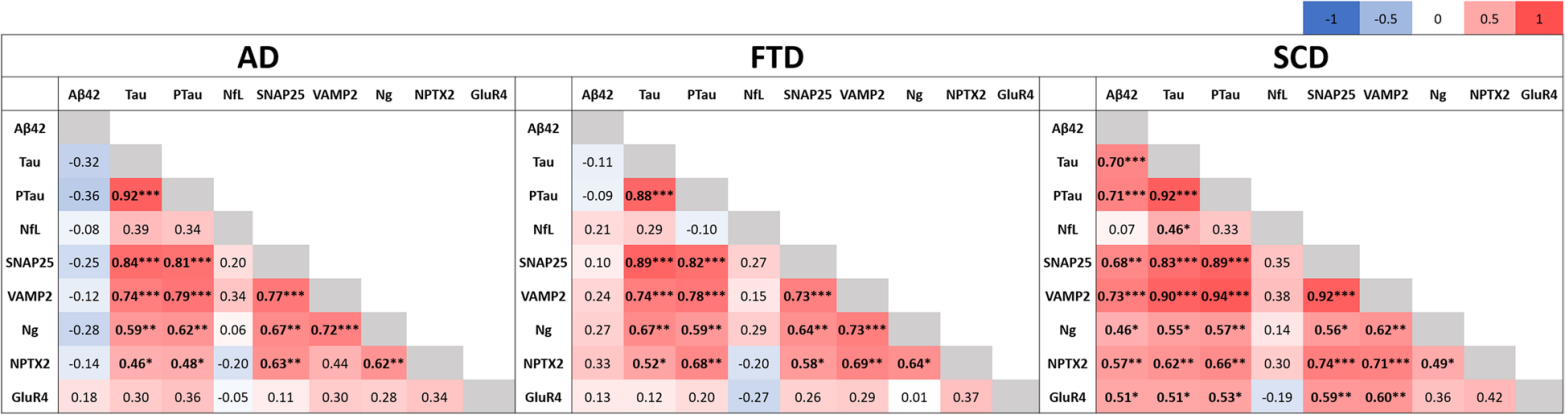
Correlation matrix of the CSF biomarkers. Pearson’s correlation was used as all the biomarkers were log-transformed to fit a normal distribution. The significant correlations are indicated in bold. *P < 0.05, **P < 0.01, ***P < 0.001

The correlation matrix of the CSF biomarkers is presented in Fig. 4. In the SCD group, synaptic proteins were all well correlated (Pearson, *r* = 0.92–0.49), although no significant correlations were found between GluR4 and Ng or NPTX2. However, the strength of these correlations were reduced (Pearson, *r* = 0.73–0.58) and often not significant in the AD and FTD clinical groups. Furthermore, we did not find a correlation of the axonal biomarker NfL to any synaptic biomarker within this cohort.

The presynaptic vesicle proteins, SNAP25 and VAMP2, correlated with each other as well as with Tau, PTau, and Ng (Pearson, *r* = 0.94–0.55) in all three clinical groups. Moreover, the postsynaptic biomarker Ng correlated with SNAP25, VAMP2, and NPTX2 (Pearson, *r* = 0.73–0.49) in all the clinical groups. In the SCD group, GluR4 had a significant association with SNAP25 and VAMP2 (Pearson, *r* = 0.60–0.59), although this association was not present in the AD or FTD clinical groups. Interestingly, all CSF biomarkers (except NfL) had a significant correlation with Aβ42 in the SCD group (Pearson, *r* = 0.73–0.46), although this correlation was lost in the other clinical groups.

### Association of the synaptic biomarkers with cognitive scores of patients

**Fig. 5:**
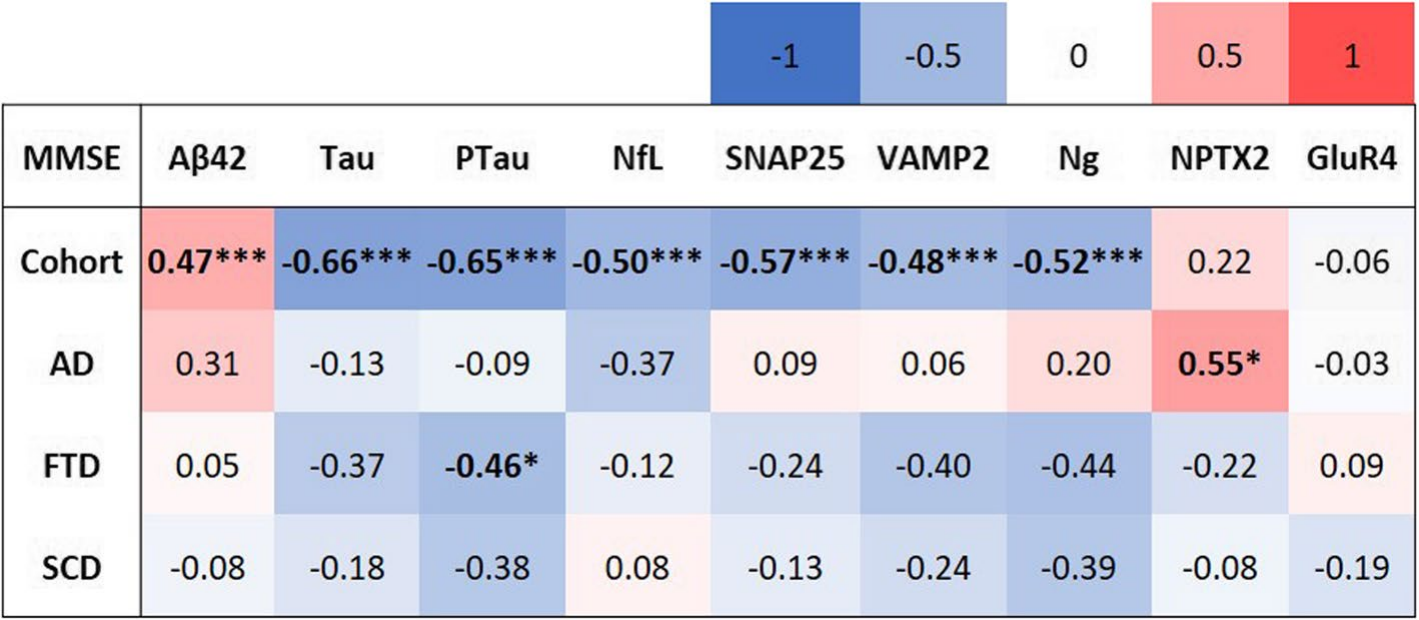
Correlation matrix of the CSF biomarkers with MMSE scores in the entire cohort and upon stratification into the clinical groups. Spearman’s correlation was used as MMSE scores were not normally distributed even after log transformation. The significant correlations are indicated in bold. *P < 0.05, **P < 0.01, ***P < 0.001

The correlation matrix of the CSF biomarkers with the cognitive (MMSE) scores of patients is presented in Fig. 5, and the scatter plots of these correlations are shown in Supplementary Fig. 8. We found that in the overall cohort, the biomarkers SNAP25 (Spearman, *r* =  − 0.57, *P* < 0.001), VAMP2 (Spearman, *r* =  − 0.48, *P* < 0.001), and Ng (Spearman, *r* =  − 0.52, *P* < 0.001) were negatively correlated with the MMSE scores of the patients, indicating that an increase in these synaptic protein concentrations in CSF was associated with decreasing cognition. Upon stratification for clinical diagnosis, NPTX2 in AD patients correlated moderately with their MMSE scores (Spearman, *r* = 0.55, *P* = 0.012). No other associations of the synaptic biomarkers with cognitive scores were detected in the stratified cohort.

## Discussion

We measured the concentration of the synaptic proteins SNAP25, VAMP2, Ng, NPTX2, and GluR4 in the CSF of patients with AD, FTD, and SCD. We have shown that both AD and FTD patients have an elevated expression of SNAP25, VAMP2, and Ng compared to SCD, while NPTX2 levels are lower in FTD compared to SCD. Herein, we also reported the development and clinical validation of two novel immunoassays for the SNARE complex proteins SNAP25 and VAMP2. Synaptic proteins involved in diverse functional aspects and localizations were assessed in this cohort as a first attempt to highlight the diversity in synaptic pathology and overlapping pathologies in patients with AD and FTD. Additionally, we observed a differential degree of correlation among the synaptic proteins in AD, FTD, and SCD, which provides proof of concept that synaptic protein levels and their co-expressions are altered in different disease states. The association of each synaptic protein with the classical fluid biomarkers Aβ42, Tau, PTau, and NfL as well as with the cognitive scores of patients was investigated.

To our knowledge, this is the first clinical cohort to report that the CSF synaptic proteins SNAP25 and VAMP2 are elevated in FTD patients and are not specific to AD. In a recent study consisting of neuropathology-confirmed FTD patients, VAMP2 was not found to be significantly increased for FTD compared to controls, although an increasing trend was detected [29]. Although Ng is primarily known to be an AD-specific biomarker, we found that Ng levels, compared to SCD patients, were also significantly elevated in both AD and FTD patients but failed to significantly distinguish these two clinical groups. Similar results have been reported in a previous cohort consisting of AD and FTD patients [30]. The plausible reasons for the lack of differential CSF profiles of synaptic proteins in the two disease groups could be the heterogeneity of FTD as a disease, its co-pathologies with AD and other dementias, or the sheer underrepresentation of FTD in clinical cohorts due to misclassification [10, 31–33]. Furthermore, it has been suggested that the implied AD specificity of Ng could be a result of selection bias in cohorts. This is because clinical samples are often stratified based on their Tau levels, and Ng is a biomarker that consistently correlates to high Tau and PTau levels, as we see in our cohort too [34].

NPTX2 reportedly has predictive value for patients with genetic FTD [33, 34]. In this cohort, the NPTX2 levels were significantly lower in FTD patients compared to SCD. However, the FTD patients were not stratified based on their genetic diversity or disease severity, and the lack of differential expression of NPTX2 between AD and FTD could possibly be due to the heterogeneity of these FTD patients. Another notable outcome was that the CSF GluR4 levels were comparable across the clinical groups in this cohort. GluR4 was recently identified as a biomarker that demonstrates preclinical changes in AD patients before neurodegeneration and Tau pathology, although the diagnostic and prognostic value of this AMPA receptor complex protein remains to be evaluated in depth [5, 7]. Given that a cohort reported elevated GluR4 levels in prodromal AD patients compared to controls, it is plausible that the difference is less prominent in this cohort due to the demented stage of AD disease progression [5]. SNAP25 had the highest diagnostic value between AD versus SCD groups and was also the only biomarker selected to form the differential diagnostic panel between these groups. Furthermore, SNAP25 consistently had the highest AUC among individual synaptic proteins between the FTD versus SCD as well as AD versus FTD patients. Interestingly, for the differential diagnosis of FTD versus SCD, SNAP25 was not selected as a part of the differential biomarker panel which consisted of VAMP2 and NPTX2. Between AD versus FTD patients, the differential diagnostic panel consisted of the two presynaptic SNARE proteins SNAP25 and VAMP2.

We found that SNAP25, VAMP2, Ng, and NPTX2 correlated significantly with CSF Tau and PTau in all three clinical groups. GluR4 correlated with Tau and PTau markers in SCD alone. While all synaptic proteins correlated with CSF Aβ42 in SCD, there was no correlation found between these biomarkers in AD and FTD patients. A strong positive correlation between SNAP25 and VAMP2 was also found throughout all the clinical groups, which could be explained by the shared mechanistic pathways of these two SNARE proteins [15]. Furthermore, we found that there was a strong correlation between SNAP25 and VAMP2 with Ng, though these proteins localize differently at the synapse. Post-synaptic protein Ng correlated moderately but significantly with NPTX2 in each clinical group, although there were no significant correlations detected between Ng and GluR4.

These results are in line with a previous observation that synaptic proteins in the brain are differently involved in pathological disease states [9]. Likewise, there is a positive correlation between Aβ42 and all other synaptic biomarkers in the SCD group, but the absence of this correlation in FTD and negative (non-significant) correlation factors in AD patients is of note. Moreover, the association of GluR4 with all CSF biomarkers (except NfL) in the SCD group but not in the AD and FTD clinical groups stands out. This absence of correlation of GluR4 in disease states could be an indicator of lack of normal physiology and its possible use as a biomarker for disease prognosis at early stages [5, 29]. Of particular note is the evidence that no synaptic proteins were not associated with CSF NfL in any clinical group, concurrent with data from other recent cohorts [1, 9, 29]. This observation is of interest for understanding the mechanisms behind synaptic pathology and axonal degeneration which may be distinct.

In this cohort, the synaptic biomarkers SNAP25, VAMP2, and Ng showed significant negative correlations with the cognitive scores of patients. An increase in CSF synaptic protein levels indicated worsening cognitive health of patients and progressive neurodegeneration, as expected. These associations were, however, lost upon stratification for their clinical diagnosis, maybe due to the low sample size in each group as well as overlapping pathologies.

The contradictory and cohort-specific reports about the correlation between CSF synaptic proteins not only reflect the complexity of synaptic pathology, but also a more fundamental issue of differential clinical characterization processes [8, 9, 11]. Herein, we found proof of concept that synaptic pathology is diverse and individual synaptic proteins reflect different aspects of synaptic dysfunction, which is distinct from broad-spectrum neurodegeneration as reflected by changes in Tau, PTau, and NfL. Although limitations of this cohort could arise from the small sample size of each clinical group as well as the heterogeneity within the FTD group, our findings warrant further explorations of synaptic proteins in larger and more diverse clinical cohorts, using immunoassay technologies.

## Conclusions

This pilot study indicated that the synaptic biomarkers SNAP25, VAMP2, and Ng were not specific for AD, as they were also elevated in the heterogeneous FTD group compared to SCD. The stronger correlations of the CSF biomarkers in the SCD group compared to the disease groups implied distinct synaptic pathology that may be characteristic of the AD and FTD disease states. Our study also re-iterated recent findings suggesting that CSF synaptic biomarkers reveal neuropathologies that are possibly distinct from broad-spectrum axonal degeneration as exhibited by NfL. In conclusion, these diverse synaptic biomarkers hold promise for understanding the pathophysiological states of different dementias.

## Supplementary Information

**Additional file 1.** Supplementary Tables. Supplementary Figures.

## Data Availability

Anonymized data can be made available upon reasonable request and upon consultation with the involved biobanks. Additional datasets generated during and/or analyzed during the current study are available from the corresponding author upon reasonable request.

## Abbreviations

Aβ: Amyloid beta
AD: Alzheimer’s disease
CSF: Cerebrospinal fluid
FTD: Frontotemporal dementia
GluR4: Glutamate ionotropic receptor-4
MMSE: Mini-mental state examination
NfL: Neurofilament light
Ng: Neurogranin
NPTX2: Neuronal pentraxin-2
PTau: Phosphorylated Tau
SCD: Subjective cognitive decline
SNAP25: Synaptosomal associated protein-25
Simoa: Single molecular array
Tau: Total Tau
