# Supplementary material for: Synaptic biomarkers in the cerebrospinal fluid associate differentially with classical neuronal biomarkers in patients with Alzheimer’s disease and frontotemporal dementia
Source: Alzheimers Res Ther. 2023 Mar 24;15:62. doi: 10.1186/s13195-023-01212-x (PMC10037899; doi:10.1186/s13195-023-01212-x)
Supplement: Supplementary file 1 — Additional file 1. Supplementary Tables. Supplementary Figures. [file 13195_2023_1212_MOESM1_ESM.docx]

***SUPPLEMENTARY FILE***

**ANTIBODIES USED FOR DEVELOPMENT OF NOVEL IMMUNOASSAYS**

**VAMP2 ELISA:** The two antibodies used to develop the VAMP2 immunoassay recognized non-overlapping sequences of human VAMP2 (Uniprot ID P63027). G11 was used as the capture antibody and reacted with the synthetic peptides (Pt 1, Pt 2, and Pt 3). The detector antibody 15E4 showed reactivity with Pt 4, Pt 5, Pt 6, and Pt 7 (Supplementary Figure 1, Supplementary Table 1). The western blot (Supplementary Figure 2 A, B) revealed that the antibody 15E4 cross-reacted with recombinant human VAMP1 (rhVAMP1) along with rhVAMP2, while G11 appeared to be specific for rhVAMP2 in this blot. Neither antibody reacted with rhVAMP3. Both antibodies reacted strongly with the whole soluble brain (h181 and h1389) and synaptosome fractions (h181syn).

**SNAP25 SIMOA:** The epitope recognition sites of the antibodies against human SNAP25 are shown in Supplementary Figure 3 and Supplementary Table 2. From this data, we concluded that the ADx404 mapped epitopes in the extreme N-terminus of the SNAP25 protein, while the antibody RD042 mapped between the amino acids 24 to 39 from the human SNAP25 sequence (Uniprot ID P60880). ADx404 reacted with the synthetic peptide sequences Pt 1, 2, and acetylated Pt 5, while RD042 showed reactivity to Pt 3 and 4. The two antibodies recognized non-overlapping peptide sequences of the SNAP25 protein. The western blot in Supplementary Figure 4 shows the reactivity of the antibodies ADx404 and RD042 with full-length rhSNAP25 and whole soluble brain (h181 and h1389) and synaptosome fractions (h181syn). Both ADx404 and RD042 antibodies recognized native SNAP25 in the human brain and thus, showed strong reactivity with h181, h181-syn, and h1389, although the blot for ADx404 (Supplementary Figure 4. A) showed some aspecificity and/or degradation products. However, unlike RD042, ADx404 did not recognize rhSNAP25 as the epitope recognition site was present as an internal sequence without acetylation and this antibody requires the presence of acetylation for epitope recognition.(1)

| **Supplementary Table 1** | **OD (450 nm- 360 nm)** | | | | | | |
| --- | --- | --- | --- | --- | --- | --- | --- |
| **Antibody** | **Pt 1** | **Pt 2** | **Pt 3** | **Pt 4** | **Pt 5** | **Pt 6** | **Pt 7** |
| **G11 Rabbit mAb** | 3,29 | 3,33 | 2,09 | 0,01 | 0,01 | 0,01 | 0,01 |
| **15E4 Mouse mAb** | 0,01 | 0,01 | 0,02 | 1,21 | 3,32 | 3,35 | 2,19 |

***Supplementary Table 1:*** ***Reactivity of the synthetic peptides with antibodies G11 and 15E4.*** *The OD is color-coded based on the strength of the peptide reaction; the darkest indicates the strongest reactivity. (Pt: Peptide)*
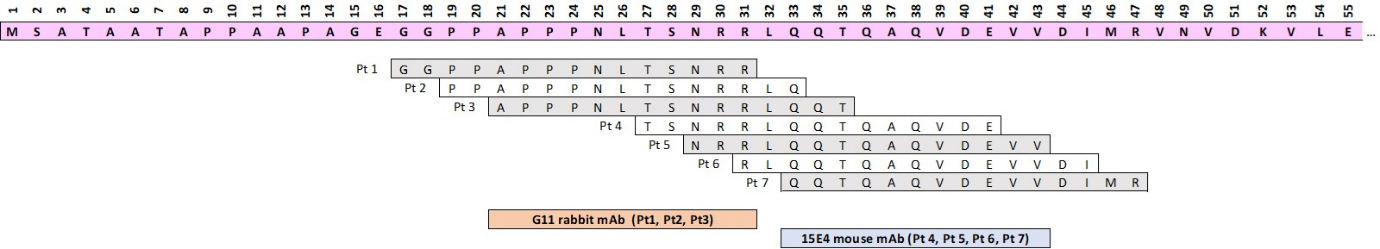


**Supplementary Figure 1:** **Epitope map of antibodies used in the VAMP2 sandwich ELISA.** G11 was used as the capture antibody and 15E4 as the biotinylated detector antibody. (Pt: Peptide)


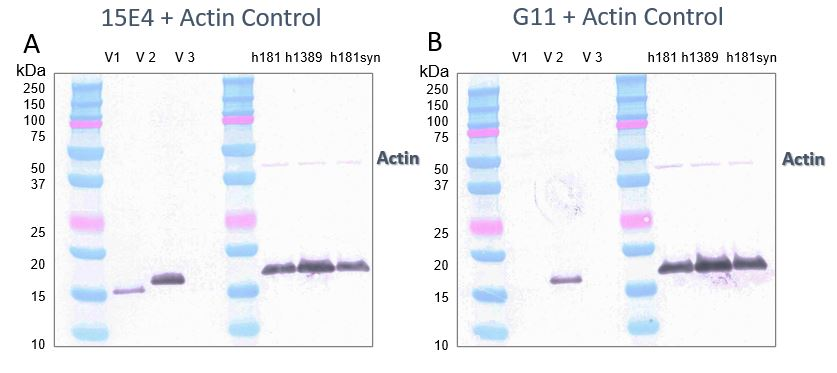


**Supplementary Figure 2:** **Specificity of the antibodies used to develop the VAMP2 ELISA.** The specificity was tested on purified recombinant human VAMP1, VAMP2, and VAMP3 (V1, V2, V3) proteins as well as the human brain (h181, h1389) and human synaptosome homogenates (h181syn). A) 15E4 mouse mAb, B) G11 Rabbit mAb. An anti-actin antibody was used as a positive loading control for the brain extracts h181, h1389, and the human synaptosome homogenate, h181syn.

| **Supplementary Table 2** | **OD (450 nm- 360 nm)** | | | | |
| --- | --- | --- | --- | --- | --- |
| **Antibody** | **Pt1** | **Pt2** | **Pt3** | **Pt4** | **Pt5** |
| **ADx404 Mouse mAb** | 3,21 | 3,31 | 0,05 | 0,03 | 3,30 |
| **RD042 Mouse mAb** | 0,05 | 0,03 | 0,22 | 0,26 | 0,12 |

***Supplementary Table 2:*** ***OD values of reactivity of the synthetic peptides with antibodies ADx404 and RD042.*** *The OD is color-coded based on the strength of the peptide reaction; the darkest indicates the strongest reactivity. (Pt: Peptide)*


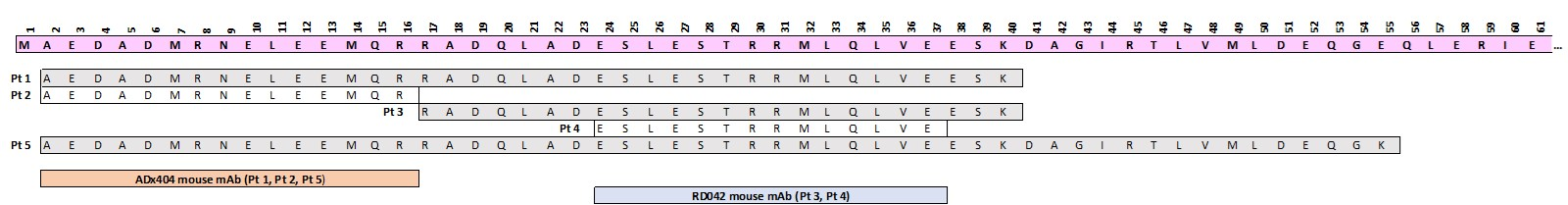


**Supplementary Figure 3**: **Epitope map of antibodies used in SNAP25 Simoa.** ADx404 was used as the capture antibody and biotinylated RD042 as the detector. Pt 5 is acetylated. (Pt: Peptide)


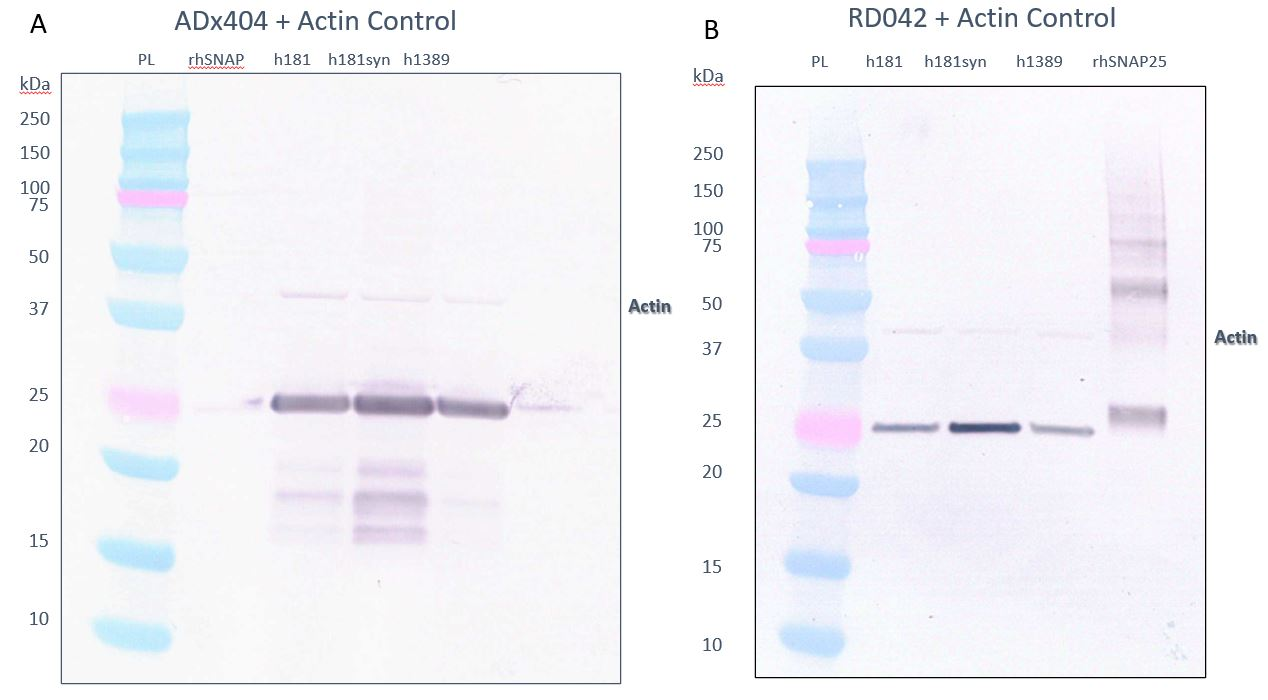


**Supplementary Figure 4:** **Specificity of the antibodies used to develop the SNAP25 Simoa.** The specificity was tested on purified recombinant human SNAP protein (rhSNAP) as well as the human brain and human synaptosome homogenates. A) ADx404 mouse mAb, B) RD042 mouse mAb. An anti-Actin antibody was used as a positive loading control for the human brain extracts h181, h1389, and the human synaptosome homogenate h181syn.


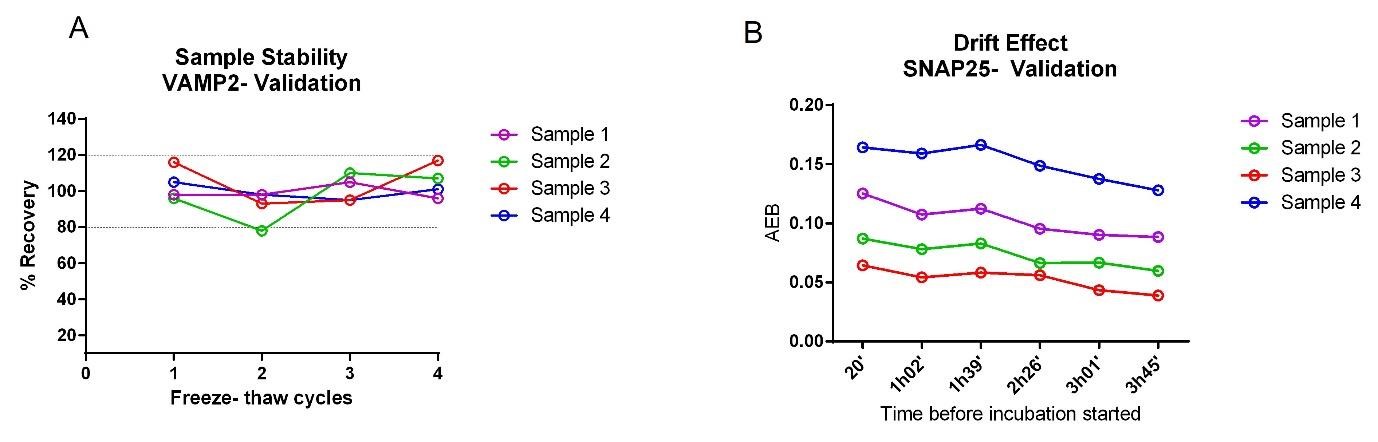


***Supplementary Figure 5: Additional Validation parameters for the two novel immunoassays.*** *A) Sample stability for VAMP2 ELISA. The CSF samples remain stable up to 4 cycles of freeze/ thaw. B) Drift effect for SNAP25 Simoa. There is a negative drift effect observed with increase in CSF incubation time. It is therefore recommended to limit the incubation time to 1 hour and 30 minutes for this immunoassay.*


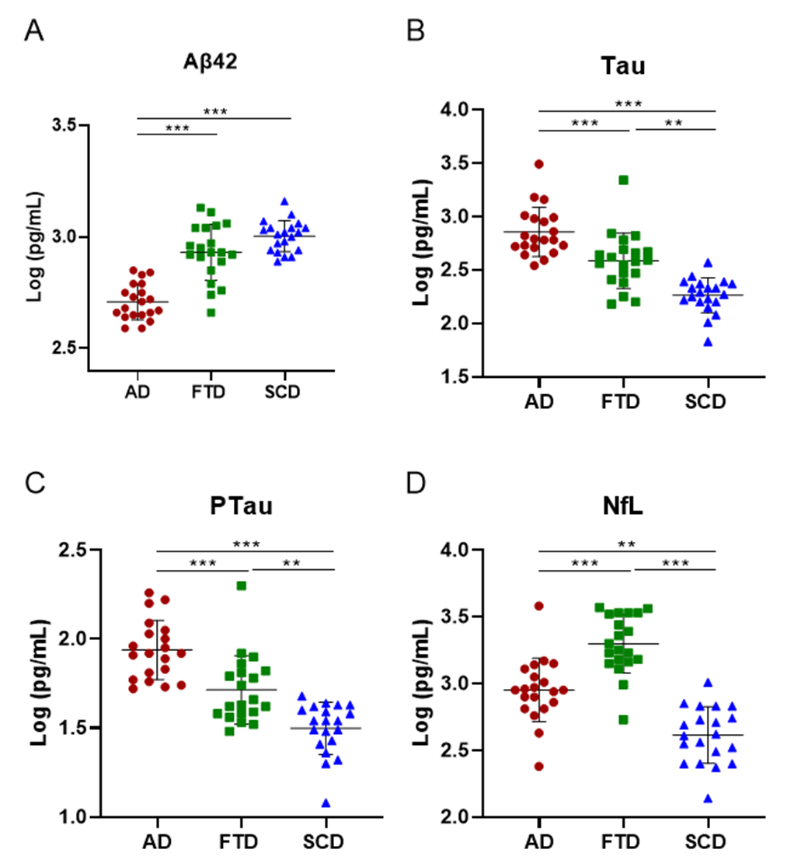


***
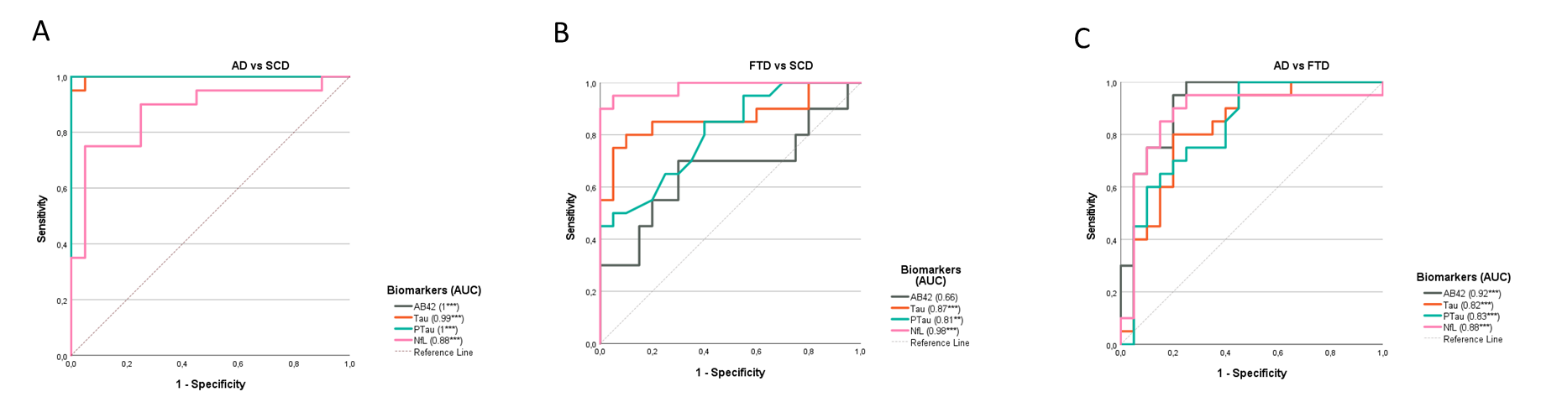
Supplementary Figure 6: Scatter plot of the core CSF fluid biomarkers in the three diagnostic groups AD (n=20), FTD (n=20) and SCD (n=20).*** *The biomarker values were log transformed to fit a normal distribution and the model corrected for age and sex prior to conducting pairwise multiple comparisons. A) AB42, B) Tau, C) pTau, D) NfL. *P<0.05, **P<0.01, ***P<0.001.*

***Supplementary Figure 7: ROC Curves and AUC values of the core CSF biomarkers amongst the clinical groups.*** *A) AD versus SCD. B) FTD versus SCD. C) AD versus FTD. (AUC: Area under the curve).* **P<0.05, **P<0.01, ***P<0.001.*


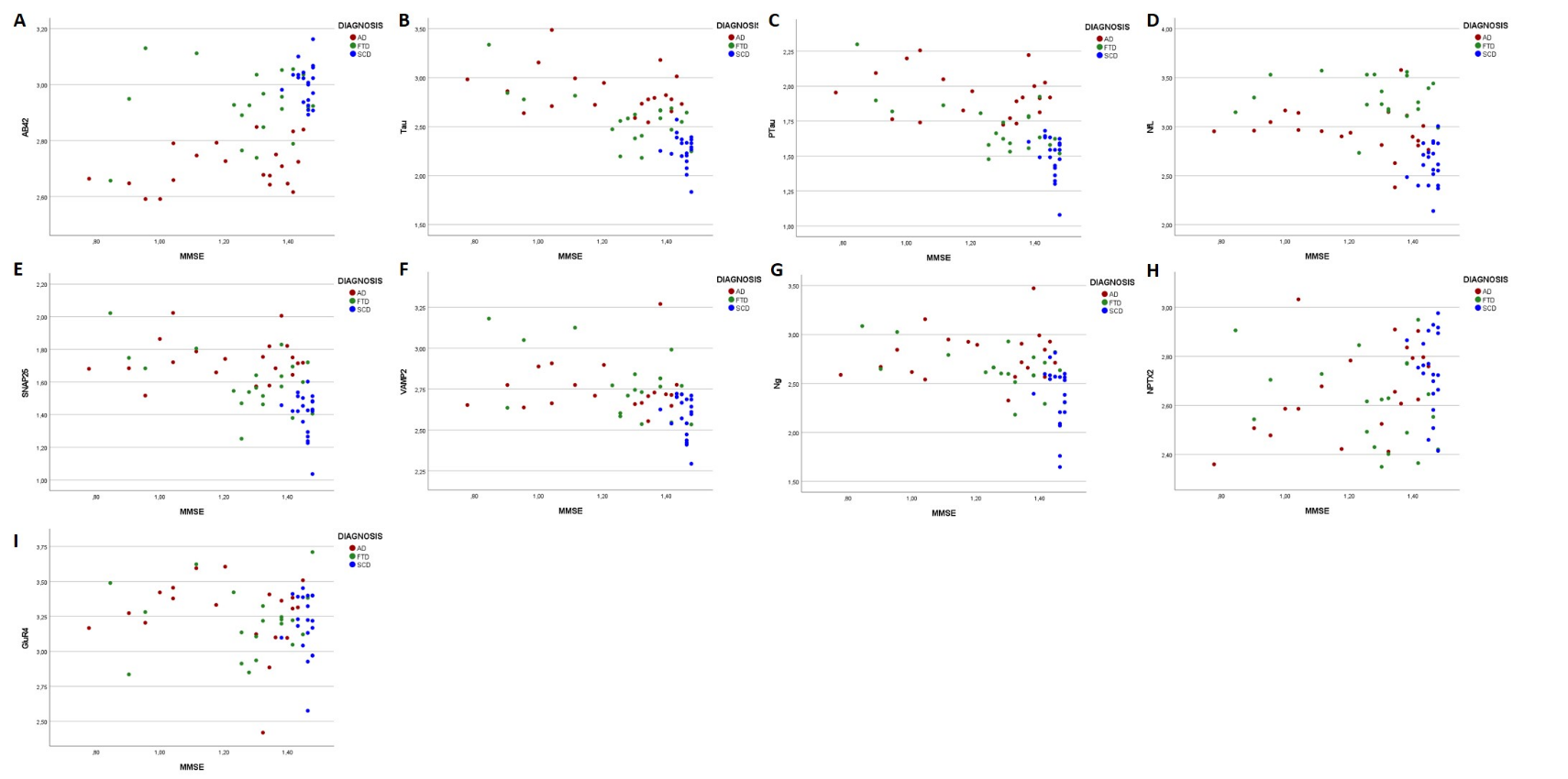


**Supplementary Figure 8: Scatter plots showing the correlation between CSF biomarkers and MMSE scores.** A) Aβ42 vs MMSE (Spearman, r= 0.47***). B) Tau vs MMSE (Spearman, r=-0.66***. C) PTau vs MMSE (Spearman, r=-0.65***). D) NfL vs MMSE (Spearman, r=-0.50***). E) SNAP25 vs MMSE (Spearman, r=-0.57***). F) VAMP2 vs MMSE (Spearman, r=-0.48***). G) Ng vs MMSE (Spearman, r=-0.52***). H) NPTX2 vs MMSE (Spearman, r=0.22). I) GluR4 vs MMSE (Spearman, r=-0.06). *P<0.05, **P<0.01, ***P<0.001.

**References**

1. Connell E, Darios F, Peak-Chew S, Soloviev M, Davletov B. N-terminal acetylation of the neuronal protein SNAP-25 is revealed by the SMI81 monoclonal antibody. Biochemistry. 2009;48(40):9582-9.
